# Inhibition of Acetyl-CoA Carboxylase 1 (ACC1) and 2 (ACC2) Reduces Proliferation and *De Novo* Lipogenesis of EGFRvIII Human Glioblastoma Cells

**DOI:** 10.1371/journal.pone.0169566

**Published:** 2017-01-12

**Authors:** Jessica E. C. Jones, William P. Esler, Rushi Patel, Adhiraj Lanba, Nicholas B. Vera, Jeffrey A. Pfefferkorn, Cecile Vernochet

**Affiliations:** Cardiovascular, Metabolic, and Endocrine Diseases (CVMED) Research Unit, Pfizer Inc, Cambridge, Massachusetts, United States of America; University of Mississippi, UNITED STATES

## Abstract

Tumor cell proliferation and migration processes are regulated by multiple metabolic pathways including glycolysis and de novo lipogenesis. Since acetyl-CoA carboxylase (ACC) is at the junction of lipids synthesis and oxidative metabolic pathways, we investigated whether use of a dual ACC inhibitor would provide a potential therapy against certain lipogenic cancers. The impact of dual ACC1/ACC2 inhibition was investigated using a dual ACC1/ACC2 inhibitor as well as dual siRNA knock down on the cellular viability and metabolism of two glioblastoma multiform cancer cell lines, U87 and a more aggressive form, U87 EGFRvIII. We first demonstrated that while ACCi inhibited DNL in both cell lines, ACCi preferentially blunted the U87 EGFRvIII cellular proliferation capacity. Metabolically, chronic treatment with ACCi significantly upregulated U87 EGFRvIII cellular respiration and extracellular acidification rate, a marker of glycolytic activity, but impaired mitochondrial health by reducing maximal respiration and decreasing mitochondrial ATP production efficiency. Moreover, ACCi treatment altered the cellular lipids content and increased apoptotic caspase activity in U87 EGFRvIII cells. Collectively these data indicate that ACC inhibition, by reducing DNL and increasing cellular metabolic rate, may have therapeutic utility for the suppression of lipogenic tumor growth and warrants further investigation.

## Introduction

Cancer cells frequently exhibit alterations in their metabolic capacity and proliferation which is accompanied by significant changes in lipids synthesis, transport and metabolism. In 1926, Otto Warburg made the observation that cancer cells produce most of their ATP via glycolysis and thus would rely to a lesser extent on mitochondrial oxidative phosphorylation (OXPHOS) [1]. Aerobic glucose utilization as well as fatty acid precursors, amino acids, nucleotides and signaling molecules provides a constant energy supply, that is essential for cancer cell growth and proliferation [2]. Many tumor tissues, such as mammary [3], prostate [4], ascites [5] and hepatic [3,6] have been shown to have a high rate of de novo lipogenesis (DNL), irrespective of the exogenous supply of lipids available in the circulation. The lipogenic switch is thought to be a mechanism to provide significant lipids building capacity in order to attain rapid expansion as well as potentially serve as a supply of nutrients [7]. This cell autonomous generation of lipids provides an independence from systemic regulation and, through intervention in this synthetic pathway, can also offer a novel therapeutic strategy to treat lipogenic cancers [8]. Modulation of lipogenic genes or proteins such as ATP citrate lyase (ACLY) [9], acetyl-coA carboxylase (ACC) [10–12] and fatty acid synthase (FASN) [13,14] either by chemical inhibitors or by RNAi-mediated gene silencing studies have been successful in demonstrating decreased cell proliferation and increased apoptosis in cancer models both *in vitro* and *in vivo*.

Acetyl-CoA carboxylases (ACC) are rate-limiting enzymes involved in the de novo synthesis of fatty acids by catalyzing the ATP-dependent carboxylation of acetyl-CoA to generate malonyl-CoA, which is then further converted to long-chain fatty acids by fatty acid synthase (FASN) [15,16]. Two isoforms of ACC encoded by different genes have been described, ACC1 and ACC2, which differ by their cellular location, with ACC1 located in the cytosol and ACC2 being associated with the mitochondrial membrane [17]. Recent studies have reported an up-regulation of ACC1 in multiple human cancers, most likely to promote lipogenesis and meet the need for rapid growth and proliferation [18–20].

Glioblastoma multiforme (GBM) is the most common brain tumor and is a markedly aggressive cancer associated with a very poor prognosis [21,22]. The most common epithelial growth factor receptor (EGFR) mutation associated with GBM is the epithelial growth factor receptor mutant III (EGFRvIII), characterized by the deletion of exons 2–7 leading to a constitutively active receptor [21]. To our knowledge, this mutation has not been detected in normal tissue [22]. The EGFRvIII mutation is known to reprogram tumor cell metabolism, characterized by enhanced glycolysis [23–25], lipogenesis [26] and tumorigenic capacity offering a significant growth advantage compared to wild type EGFR, as well as protection from apoptosis [21,24].

We aimed to characterize U87 EGFRvIII cells proliferation, lipid and bioenergetic profiles and to investigate the impact of our dual ACC1/ACC2 inhibitor (ACCi) [27] on those parameters. By comparison to U87 wild type EGFR cells, we were able to demonstrate that U87 EGFRvIII cells have a higher proliferation and metabolic capacity. Importantly, while ACCi treatment blunted DNL in both cell lines, it drastically impaired U87 EGFRvIII cell proliferation and triggered cell death by apoptosis, thus highlighting ACCi as a potential therapy for GBM and perhaps other types of lipogenic cancers.

## Materials and Methods

### Reagents

Carbonyl cyanide 4-(trifluoromethoxy)phenylhydrazone (FCCP), antimycin A, rotenone, glucose, oligomycin, 2-deoxyglucose, L-carnitine, sodium palmitate, ultra fatty acid-free bovine serum albumin (FAF-BSA), Tween-20 and DMSO were obtained from Sigma (MO, USA). High glucose DMEM, fetal bovine serum (FBS), charcoal-striped fetal bovine serum (c/s FBS), L-glutamine, penicillin/streptomycin, geneticin (G418), SuperSignal West Fempto maximum sensitivity substrate, RIPA lysis and extraction buffer, TBS, PBS, mammalian protein extraction reagent (MPER), Lipofectamine RNAiMax transfection reagent and Pierce BCA and micro BCA protein assay kits were obtained from ThermoFisher Scientific (MA, USA). Seahorse XF base assay medium was obtained from Agilent Technologies (CA, USA). Antibodies and protease and phosphatase inhibitor cocktail were obtained from Cell Signaling Technology (MA, USA). CellTiter-Glo, CytoTox 96 non-radioactive cytotoxicity and Caspase-Glo 3/7 assay kits were obtained from Promega (WI, USA). Microscint E, [1-14C]-Acetic Acid sodium salt and 96 well tissue-culture treated isoplates were obtained from Perkin Elmer (MA, USA). Costar white, opaque tissue-culture treated 96 well plates were obtained from Corning (NY, USA). SDS-PAGE reagents including acrylamide gels, polyvinylidene difluoride (PVDF) membranes, SDS tris-glycine running and transfer buffers were obtained from Biorad (CA, USA). Acetyl-CoA carboxylase inhibitor #28 [27] was synthesized in-house at Pfizer, Inc (CT, USA).

### Cell lines

Human glioblastoma U87 and U87 EGFRvIII cell lines were purchased and licensed from the Ludwig Institute, Switzerland.

### Media

U87 cells were cultured with media containing high glucose DMEM, 10% FBS, 4 mM glutamine and 1% penicillin/streptomycin. U87 EGFRvIII cells were cultured with media containing high glucose DMEM, 10% FBS, 4 mM glutamine, 1% penicillin/streptomycin and 0.5 mg/mL geneticin (G418). For inhibition of de novo lipogenesis and cell proliferation assays, 5% c/s FBS replaced 10% FBS. All other media components and concentrations remained the same. For cellular bioenergetics and substrate utilization analysis, Seahorse Biosciences XF base assay medium containing 0.8 mM MgSO_4_, 1.8 mM CaCl_2_, 143 mM NaCl, 5.4 mM KCl, 0.91 mM NaH_2_PO_4_, 3mg/mL Phenol Red and amino acids was used and supplemented as indicated in the experimental method as well as in S1 and S2 Tables.

### Preparation of Bovine Serum Albumin (BSA)-conjugated palmitate

Briefly, a 150 mM NaCl solution was warmed in a 37°C water bath. 11.34g of FAF-BSA was added to 100 mL of warmed 150 mM NaCl solution in a beaker. The beaker was then covered with parafilm and placed into a 37°C water bath, with constant stirring until the FAF-BSA was dissolved. Then the FAF-BSA solution was filter sterilized and 50mL transferred to a clean beaker in 37°C water bath with constant stirring for conjugation to palmitate. The remaining 50 mL of FAF-BSA was diluted with 50 mL 150 mM NaCl and aliquoted into glass vials for use as a negative control (0.85 mM FAF-BSA). Palmitate solution was prepared by adding 153 mg of sodium palmitate to 44mL of 150 mM NaCl solution in a beaker. The beaker was then placed into a 70°C water bath with constant stirring until the palmitate dissolved and the solution became clear. To conjugate the palmitate to FAF-BSA, 40 mL of the palmitate solution was slowly transferred into 50mL of FAF-BSA solution at 37°C with constant stirring. Once the transfer was complete, the solution was maintained at 37°C with constant stirring for 1 hour. After which, the final volume was adjusted to 100mL with 150 mM NaCl solution (5 mM palmitate-BSA). The pH was adjusted to 7.4 with 1N NaOH. Finally, the solution was sterilized by filtration and aliquoted into glass vials before being stored at -20°C.

### Real-time quantitative PCR with reverse transcription

RNA was isolated from U87 and U87 EGFRvIII cells using a Qiagen RNeasy kit (Qiagen, Germany). mRNA was reverse transcribed to cDNA using iScript cDNA synthesis kit (Bio-Rad, USA). The abundance of transcripts was assessed by real-time PCR on a C1000 Thermal Cycler (BioRad, USA). The expression data for the genes of interest were normalized to the expression of housekeeping genes (*hPPIA* and *hTBP*). Taqman primers for each gene were obtained from ThermoFisher Scientific (, MA, USA) and are listed in S3 Table.

### Western blot analysis

Total proteins were extracted from cultured cells through the addition of RIPA lysis and extraction buffer containing a cocktail of protease and phosphatase inhibitors to each well followed by scraping the cell monolayer from the culture well and transferring to a fresh microcentrifuge tube. The cell suspension was then incubated on ice for 15 minutes with periodic mixing to aid cell lysis. The cell lysate was then cleared of insoluble proteins by centrifugation at 14,000 rpm for 15 minutes at 4°C. The resulting supernatant was transferred to a clean microcentrifuge tube and protein concentration was measured using a Pierce BCA protein kit. For ACC1 and ACC2 protein analysis, 12 μg total proteins were subjected to SDS-PAGE. Proteins were transferred to a polyvinylidene difluoride (PVDF) membrane. Membranes were blocked for 1 hour at room temperature in 5% fat-free milk in TBS buffer supplemented with 0.2% Tween-20 (TBS-T) with constant agitation. After a brief wash with TBS-T, membranes were incubated overnight at 4°C with primary antibodies diluted 1:1000 in 5% BSA/TBS-T. Following incubation with primary antibodies, membranes were washed with TBS-T and then incubated with a HRP-linked goat anti-rabbit IgG secondary antibody diluted 1:5000 in 5% BSA/TBS-T and incubated for 1 hour at room temperature with constant agitation. Membranes were visualized by using SuperSignal West Fempto substrate and BioRad ChemiDoc imaging system (Bio-Rad, USA). Quantification of band volume intensity was performed using ImageLab software (BioRad, USA). A table of the antibodies used is provided in S4A Table.

### Knockdown of ACC1 and ACC2 with siRNA treatment

U87 and U87 EGFRvIII cells were plated with 5% c/s FBS media at 150,000 cells/well into 6 well plates and incubated overnight in a humidified incubator at 37°C/5% CO_2_. The following day, the cells were treated with a combination of 20 nM siRNA targeted to ACACA (ACC1) and 20 nM siRNA targeted to ACACB (ACC2) or identical concentration of scrambled control siRNA formulated into lipid complexes using Lipofectamine RNAiMax transfection reagent according to manufacturer instructions for 72 or 144 hours. For the 144 hours time point, both media and siRNA complexes were refreshed after 72 hours. Specific siRNAs used are listed in S4B Table.

### Inhibition of de novo lipogenesis

U87 and U87 EGFRvIII cells were plated with 5% c/s FBS medium at 7,500 cells/well in tissue-culture treated 96 well Perkin Elmer isoplates and incubated in a humidified incubator at 37°C/5% CO_2_ for 48 hours. On day 3, ACC inhibitor (ACCi) or DMSO was added to the appropriate wells and returned to the incubator for 1 hour. After 1 hour, 0.2 μCi ^14^C-acetate was added to the appropriate wells and the plates were returned to the incubator for 5 hours. To stop the assay, the media was gently aspirated and the cell layer was washed 3 times with ice-cold PBS. Cells were lysed by the addition of 40 μL of MPER to each well and the plate was shaken for 30 minutes at room temperature, after which 160 μL Microscint E was added to each well and gently mixed. The plates were evaluated for ^14^C counts in the organic lipid phase with a Microbeta Trilux plate reader (Perkin Elmer, USA).

### Inhibition of cell proliferation

U87 and U87 EGFRvIII cells were plated with 5% c/s FBS medium at 150,000 cells/well in 6 well plates and incubated overnight in a 37°C/5% CO_2_ humidified incubator. The following day, ACC inhibitor (ACCi) or DMSO was added to the appropriate wells and the plates were returned to the incubator for 72 and 144 hours. At each time point, total protein content was measured using a Pierce BCA assay kit (Thermo Scientific) as per manufacturer instructions. For the 144HR time point, media and treatments were refreshed after 72 hours. Cell proliferation was also assessed through the measurement of total cellular ATP content using a CellTiter-Glo assay kit (Promega, USA) as per manufacturer instructions.

### Measurement of cellular bioenergetics and substrate utilization

Oxygen consumption rates (OCR) and extracellular acidification rates (ECAR) were measured using the XF96e extracellular flux analyzer (Agilent Technologies, USA) as previously described [2]. U87 and U87 EGFRvIII cells were plated into T75 flasks with 5% c/s FBS medium and incubated overnight in a 37°C/5% CO_2_ humidified incubator. The following day, the media was gently aspirated and replaced with 5% c/s FBS medium containing 30 μM ACC inhibitor (ACCi) or DMSO and returned to the incubator for 0, 72, or 144 hours. The day before the end of the treatment, U87 and U87 EGFRvIII cells were removed from the T75 flasks and plated at 20,000 cells/well into XF96e assay plates using their conditioned treatment media and returned to the incubator. For cellular bioenergetics analysis, the following day the treatment media was aspirated and replaced with Seahorse XF base assay media supplemented with 25 mM glucose and 30 μM ACCi or DMSO control. The plate was incubated in an open air incubator at 37°C for 1 hour. 10x concentrations of oligomycin (20 μM), FCCP (5 μM) as well as rotenone (10 μM) combined with antimycin A (10 μM) were loaded into the XFe96 cartridge ports A-C, respectively. After cartridge equilibration, the assay plate was loaded into the instrument to begin the assay. Each cycle consisted of 3 minutes mixing and 3 minutes of measurement. Four basal rate measurements were taken before injection of ports A and B with four measurement cycles following each injection. Three measurement cycles were recorded after injection of port C. For substrate utilization analyses, treatment media was aspirated and replaced with Seahorse XF base assay media supplemented with 0.5 mM carnitine and 30 μM ACCi or DMSO control. The plate was incubated in an open air incubator at 37°C for 45 minutes. After which, 40 μM etomoxir was added to the appropriate wells and the assay plate was returned to the 37°C open air incubator for 15 minutes. 10x concentrations of glucose (100 mM), 2-deoxyglucose (50 mM) and rotenone (10 μM) combined with antimycin (10 μM) were loaded into the XFe96 cartridge ports A-C, respectively. After cartridge equilibration and immediately prior to assay start, 100 μM palmitate or BSA control was added to the appropriate wells of the XF96e assay plate. The assay plate was then loaded into the instrument to begin the assay. Each cycle consisted of 3 minutes mixing and 3 minutes of measurement. Four rate measurements were taken before and after each injection. Following the measurement, the rate values were normalized to total protein content per well as measured by micro BCA protein assay kit as per manufacturer instructions. ATP-coupled respiration was calculated as ((basal OCR−non-mitochondrial OCR)−(oligomycin- insensitive OCR−non-mitochondrial OCR))/(basal OCR−non-mitochondrial OCR))*100. Respiratory control ratio (RCR) was calculated as (FCCP-stimulated OCR)/(oligomycin-insensitive OCR). Media compositions including specific additives used in each experiment are listed in S1 and S2 Tables.

### Mechanism of cell death

U87 and U87 EGFRvIII cells were plated with 5% c/s media at 5,000 cells/well in tissue-cultured treated white, opaque 96 well plates and incubated overnight in a 37°C/5% CO_2_ humidified incubator. The following day, ACC inhibitor (ACCi) or DMSO was added to the appropriate wells and the plates were returned to the incubator for 72 and 144 hours. At each time point, cytotoxicity and caspase activity was evaluated using CytoTox 96 non-radioactive cytotoxicity and Caspase-Glo 3/7 assay kits as per manufacturer instructions. Measurements were normalized to total protein content per well as measured by BCA protein assay kit as per manufacturer instructions. For the 144HR time point, media and treatments were refreshed after 72 hours.

### Measurement of lipid species

U87 and U87 EGFRvIII cells were plated into 6-well plates with 5% c/s media and incubated overnight in a humidified 37°C/5% CO_2_ incubator. The following day 30 μM ACC inhibitor or DMSO was added to the appropriate wells and the plates were returned to the incubator for 144 hours. Cell media and treatments were refreshed after 72 hours. After the treatment time period, the media was aspirated from the wells and the cells were washed with PBS. The cells were then lysed in 350 μL Methanol:Water (1:1, v/v). Following lysis, lipids were extracted from 200 μL of cell lysate with 200 μL Dichloromethane:Isopropanol:Methanol (25:10:65, v/v/v) containing the following internal standards at a concentration of 200 nM: Glyceryl Triheptadecanoate, 1,2-Dinonadecanoin, Cholesteryl Heptadecanoate, 1,2-Dilauroyl-sn-glycero-3-phosphocholine, 1-Heptadecanoyl-2-hydroxy-sn-glycero-3-phosphocholine, and Palmitoyl-L-carnitine-(N-methyl-d_3_) hydrochloride. Lipid extracts were then analyzed by UPLC-MS/MS using a Waters Acquity UPLC coupled to a Sciex QTrap 5500 mass spectrometer. Lipid classes were separated by reversed phase chromatography on a Waters Acquity UPLC BEH300 C4 column, 1.7 micron, 2.1 x 50 mm. Lipid species were then analyzed on the mass spectrometer using positive ion electrospray ionization in the multiple reaction monitoring (MRM) mode. LC chromatogram peak integration was performed with Sciex MultiQuant software. All data reduction was performed with in-house software.

### Statistical analysis

All data are expressed as mean +/- s.e.m. P values less than 0.05 were considered statistically significant differences as determined by one-away ANOVA, two-way ANOVA or unpaired t-tests where appropriate. Data shown are representative of repeated experiments performed as indicated.

## Results

### Characterization of proliferation and metabolic capacity of U87 and U87 EGFRvIII cell lines

**Fig 1:**
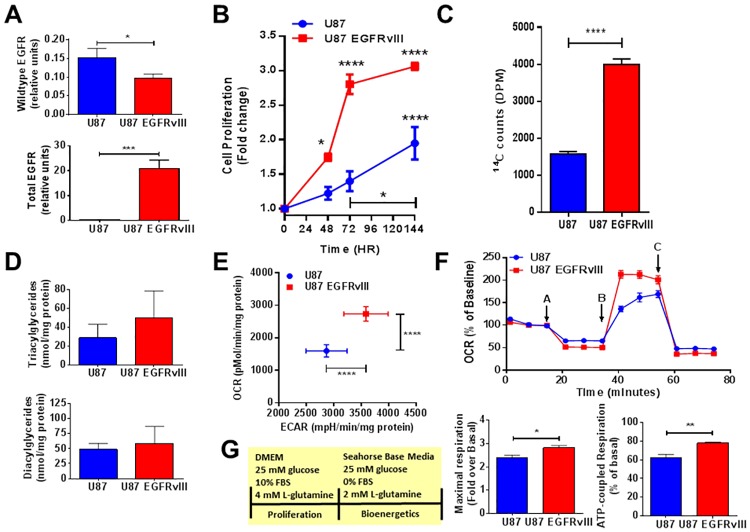
U87 EGFRvIII cells display higher proliferation, de novo lipogenesis and metabolic activity. (A) U87 EGFRvIII cells have reduced mRNA expression of WT EGFR compared to U87 cells (upper panel) and higher total EGFR including the mutated EGFRvIII expression (lower panel) as assessed by qPCR. Data are normalized to the expression of housekeeping genes. Each data point represents mean +/- sem, n = 3–4. * p<0.05, *** p = 0.0003. (B) U87 and U87 EGFRvIII cellular proliferation rates as assessed by luciferase measurement. Data are expressed as fold change from time 0 hours. Each data point represents mean +/- sem, n = 3. * p<0.05, **** p<0.0001. (C) Basal de novo lipogenesis of U87 and U87 EGFRvIII cells as assessed by measurement of the incorporation of ^14^C-acetate into neutral lipids. Data are expressed as ^14^C counts, DPM. Each data point represents mean +/- sem, n = 3. **** p<0.0001. (D) Total triacylglycerides and diacylglycerides content of U87 and U87 EGFRvIII cells at basal as assessed by UPLC-MS/MS measurement. Data are expressed as nmol/mg protein. Each data point represents mean +/- sem, n = 3. (E) Basal OCR and ECAR measurements from U87 and U87 EGFRvIII cells as assessed using Seahorse XF96e system. Data are expressed as pmol/min/mg protein (OCR) and mpH/min/mg protein (ECAR). Each data point represents mean +/- sem, n = 5. ****p<0.0001. (F) Bioenergetics profile of U87 and U87 EGFRvIII as assessed using Seahorse XF96e system. Injections A, B and C are oligomycin, FCCP and a mixture of rotenone and antimycin A respectively (top panel). Data are represented as % of baseline respiration, fold change from basal respiration and % of basal respiration. Each data point represents mean +/- sem, n = 5. *p<0.05, **p<0.01. (G) Media compositions for Fig 1E and 1F. Proliferation refers to media used for cell maintenance and growth before the bioenergetics experiments.

Glioblastoma (GBM) tumors have been shown to express both wild-type EGFR as well as mutated EGFR, with EGFRvIII being the most common mutation [21]. We found that U87 and U87 EGFRvIII cell lines express similar levels of full length wild type EGFR mRNA as quantified by real-time qPCR in Fig 1A, upper panel. What sets the U87 EGFRvIII cells apart from the U87 control cells is the addition of a constitutively active form of EGFR, EGFRvIII, which lacks exons 2–7, resulting in a significantly higher expression of total EGFR including EGFRvIII (Fig 1A, lower panel). Consistent with the aggressiveness of GBM, the U87 EGFRvIII cell line displayed significantly greater proliferation when compared to the U87 control cell line as measured by increased luciferase signal, a marker of cellular ATP content, significant at 48 hours (Fig 1B). To investigate the relationship between the de novo synthesis of lipids and cellular proliferation, we evaluated both U87 and U87 EGFRvIII cell lines in the presence of ^14^C-acetate and quantified the intracellular accumulation of ^14^C-labeled neutral lipids after 5 hours (Fig 1C). U87 EGFRvIII cellular ^14^C-acetate incorporation into neutral lipids was significantly elevated by 2.5 fold compared to U87 cells (4000 ± 142.3 versus 1566 ± 72.8 DPM), while the U87 EGFRvIII cellular number was increased by only 1.7 fold (99421 ± 2958 versus 56951 ± 2033 relative luciferase units), as measured by total ATP content (S1 Fig), therefore demonstrating a greater capacity for lipids synthesis per cell for U87 EGFRvIII cells. Based on this observation, we assessed the relative lipids composition of each cell line, U87 and U87 EGFRvIII, by ultra performance liquid chromatography and mass spectrometry (UPLC-MS/MS). Total triacylglycerides (TAG) and diacylglycerides (DAG) were found to be at similar levels in both cell lines represented in Fig 1D.

In addition, as increased cancer cell proliferation has been associated with increased metabolic rates, we investigated U87 and U87 EGFRvIII cellular bioenergetics (oxygen consumption rate (OCR) and extracellular acidification rate (ECAR)). U87 and U87 EGFRvIII cellular OCRs were assessed under a basal state and in response to various mitochondrial inhibitors or activators such as oligomycin (ATP synthase inhibitor) and FCCP (a mitochondrial activator), which allowed for the quantification of maximal mitochondrial activity as well as differentiating ATP-coupled and uncoupled OCR. Interestingly, U87 EGFRvIII cells displayed a higher baseline OCR and ECAR compared to U87 cells (Fig 1E). More importantly, when adjusted to baseline OCR, U87 EGFRvIII cells showed a 19% increase in mitochondrial maximal respiration capacity (2.822 ± 0.095 fold versus 2.375 ± 0.131 fold) as well as a 16% increase in ATP-coupled respiration (77.96 ± 0.97% versus 61.98 ± 3.83%) compared to U87 cells (Fig 1F). Taken together, these results highlight a distinct metabolic profile for U87 EGFRvIII cells compared to U87 control cells, with increased cell proliferation, lipids synthesis and a higher metabolic rate under these experimental conditions.

### Inhibition of ACC decreases de novo lipogenesis (DNL) in both U87 and U87 EGFRvIII cells and inhibits U87 EGFRvIII cell proliferation to a greater extent compared to U87 cells

**Fig 2:**
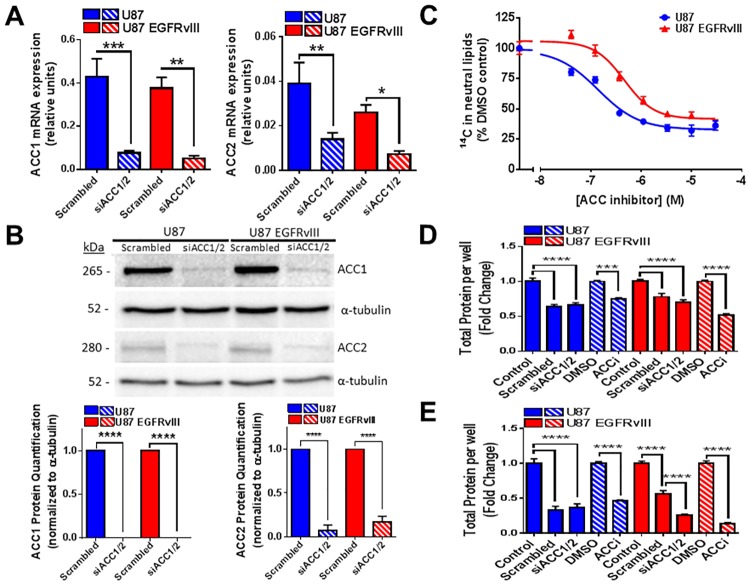
Inhibition of ACC leads to decreased de novo lipogenesis in both U87 and U87 EGFRvIII but reduced the number of U87 EGFRvIII cells to a greater extent. (A) mRNA expression of ACC1 (left panel) and ACC2 (right panel) in both U87 and U87 EGFRvIII cells after treatment with a combination of siRNAs targeted to ACC1 and ACC2 (siACC1/2) or scrambled control siRNAs as assessed by qPCR. Data are normalized to housekeeping genes expression. Each data point represents mean +/- sem, n = 3. *p<0.05, **p<0.01, *** p<0.001. (B) U87 and U87 EGFRvIII cells were treated with a combination of siRNAs targeted to ACC1 and ACC2 (siACC1/2) or scrambled control siRNAs for 144 hours. Extracted proteins were assessed by western blot (top panel). ACC1 protein (lower left panel) and ACC2 protein (lower right panel) were quantified by measurement of band intensity volume normalized to α-tubulin loading control. N = 3 experiments with representative blots shown. **** p<0.0001. (C) Inhibition of de novo lipogenesis in both U87 and U87 EGFRvIII cells as assessed by measurement of ^14^C-acetate incorporation into neutral lipids after treatment with a dose range of ACCi or DMSO control. Data are expressed as percent (%) of DMSO control. Each data point represents mean +/- sem, n = 3. (D) Total cellular proteins were assessed after 72 hours treatment with either a combination of siRNAs targeted to ACC1 and ACC2 (siACC1/2), scrambled control siRNAs, 30 μM ACCi or DMSO control. Data are represented as fold change from transfection reagent only control (Control) or DMSO control. Each data point represents mean +/- sem, n = 3. *** p<0.001, **** p<0.0001. (E) Total cellular proteins were assessed after 144 hours treatment with either a combination of siRNAs targeted to ACC1 and ACC2 (siACC1/2), scrambled control siRNAs, 30 μM ACCi or DMSO control. Data are represented as fold change from either transfection reagent only control (Control) or DMSO control. Each data point represents mean +/- sem, n = 3. *** p<0.001, **** p<0.0001.

Prior to investigating the impact of ACC inhibition on U87 and U87 EGFRvIII cells, we compared the relative mRNA expression of ACC1 and ACC2 by real-time qPCR and validated dual siRNA ACC1/ACC2 knock down efficiency in both cell lines. As shown in Fig 2A and 2B, ACC1 and ACC2 were similar in U87 and U87 EGFRvIII cells at the mRNA transcript levels (Fig 2A) and at the protein levels (Fig 2B) with ACC1 being the predominant isoform compared to ACC2 in both cell lines. Dual siRNA ACC1/ACC2 significantly reduced ACC1 and ACC2 mRNA and protein levels at 72 hours (S2A, S2B and S2C Fig) and to a greater extent at 144 hours (Fig 2A and 2B). While dual siRNA ACC1/ACC2 very efficiently knocked down ACC1 and ACC2, trace amounts of presumably active ACC1 and ACC2 proteins were still detected under these experimental conditions. To achieve nearer complete inhibition and strengthen our study regarding the role of ACC1/2 inhibition in cancer cell lines, we also performed experiments with our dual ACC1/ACC2 inhibitor (ACCi). ^14^C-acetate uptake, an indicator of DNL, was analyzed in both U87 and U87 EGFRvIII cell lines at baseline and in the presence of increasing concentrations of ACCi for 5 hours. We showed that ACCi treatment blunted ^14^C-acetate incorporation into neutral lipids in both U87 and U87 EGFRvIII cells as shown by their relatively similar IC_50_ values (U87 IC_50_ = 140 nM versus U87 EGFRvIII IC_50_ = 464 nM) in Fig 2C. To evaluate the impact of chronic ACC1/2 inhibition on U87 and U87 EGFRvIII cellular proliferation, total protein content was evaluated upon either dual siRNA ACC1/2 or in presence of ACCi for 72 and 144 hours (Fig 2D and 2E respectively). Unfortunately, scrambled control siRNA significantly decreased U87 EGFRvIII and to a greater extent U87 cell content compared to control (transfection reagent only), potentially masking the impact of dual siRNA ACC1/2 in the U87 parental cell line. Dual knockdown of ACC1/ACC2 by siRNA significantly reduced U87 EGFRvIII cellular content after 144 hours as compared to scrambled siRNA treatment but not in the U87 control cell line despite similar knockdown efficiency. Chronic incubation with ACCi reached a greater reduction of U87 EGFRvIII cellular protein content at 72 and 144 hours compared to dual siRNA knockdown of ACC1/ACC2, most likely due to residual ACC1/ACC2 expression under the experimental conditions. U87 control cellular protein content was reduced by the chronic incubation of ACCi and was not further inhibited by siRNA ACC1/2 compared to scrambled control. Altogether, U87 EGFRvIII cells displayed greater sensitivity compared to U87 control cells to the anti-proliferative effect of dual ACC1/2 inhibition. ACC1 and ACC2 mRNA expression remained unchanged over the treatment time course in both U87 and U87 EGFRvIII cells (S2D and S2E Fig) indicating that the lack of ACCi effect on U87 cell proliferation was not due to a loss of ACC expression under the experimental conditions.

### Chronic ACC inhibition alters cellular metabolism and impairs mitochondrial capacity

**Fig 3:**
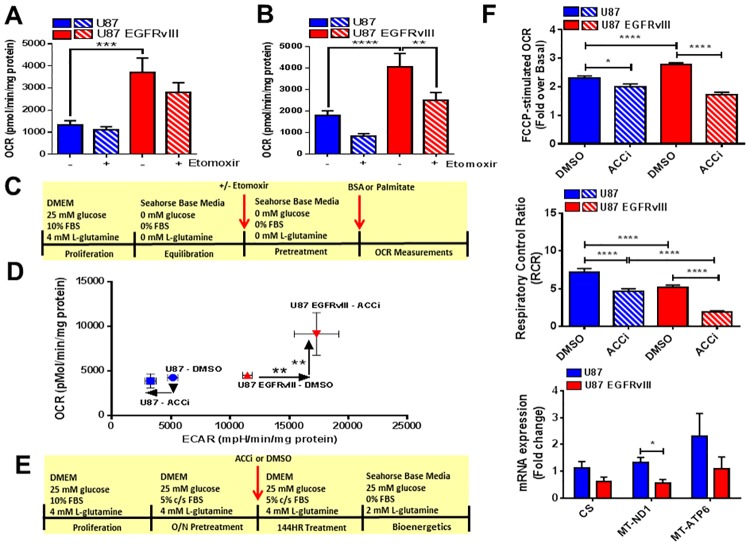
Inhibition of ACC affects mitochondrial function, gene expression and increased the glycolytic capacity of U87 EGFRvIII cells. (A) Oxygen Consumption Rate (OCR) for U87 and U87 EGFRvIII cells treated with BSA after pretreatment with either media or CPT1A inhibitor, etomoxir, or DMSO control as assessed by Seahorse XF96e system. Data are expressed as pmol/min/mg protein. Each data point represents mean +/- sem, n = 3. ***p<0.001. (B) Oxygen Consumption Rate (OCR) for U87 and U87 EGFRvIII cells treated with 100 μM palmitate after pretreatment with either media or CPT1A inhibitor, etomoxir, or DMSO control as assessed by Seahorse XF96e system. Data are expressed as pmol/min/mg protein. Each data point represents mean +/- sem, n = 3. **p<0.01, ****p<0.0001. (C) Media compositions for Figs 3A and 3B. (D) Oxygen Consumption Rate (OCR) and Extracellular Acidification Rate (ECAR) values for U87 and U87 EGFRvIII cells after 144 hours treatment with 30 μM ACCi or DMSO control as assessed by Seahorse XF96e system. Data are expressed as pmol/min/mg protein (OCR) and mpH/min/mg protein (ECAR). Each data point represents mean +/- sem, n = 3. * p<0.05, **p<0.01. (E) Media compositions for Figs 3D and 3F. (F) After 144 hours of treatment with 30 μM ACCi or DMSO control, FCCP-stimulated Oxygen Consumption Rate (OCR) (top panel), Respiratory Control Ratio (middle panel) were assessed by Seahorse XF96e system and mitochondrial gene expression (lower panel) expressed as fold change from DMSO control as assessed by qPCR. Each data point represents mean +/- sem, n = 3. * p<0.05, ****p<0.0001.

Given that the U87 EGFRvIII cell line displayed a greater mitochondrial oxidation capacity, we analyzed CPT1A expression levels, a regulator of mitochondrial fatty acid oxidation. CPT1A expression was greater in U87 control cells compared to U87 EGFRvIII cells under basal conditions (S3A Fig). At 144 hours of treatment with ACCi, CPT1A mRNA expression was significantly decreased in U87 cells but not in U87 EGFRvIII cells (S3A Fig). To address CPT1A activity in U87 and U87 EGFRvIII cell lines, CPT1A dependent respiration (OCR) was investigated in the presence and absence of etomoxir, a CPT1A inhibitor (Fig 3A) and in the presence of 100 μM palmitate (Fig 3B). Under basal conditions, U87 cellular OCR was independent of etomoxir pretreatment. Interestingly, U87 EGFRvIII cellular OCR trended down with etomoxir treatment however did not reach statistical significance (p = 0.1447) (Fig 3A). In the presence of palmitate, etomoxir reduced U87 EGFRvIII cellular OCR significantly (Fig 3B). Thus, as the basal OCR in U87 EGFRvIII cells was not affected by etomoxir treatment (p = 0.1447) and remained elevated compared to U87 cells, it is less likely that U87 EGFRvIII cells displayed greater CPT1A activity.

Since U87 and U87 EGFRvIII cell lines display significantly different proliferation responses to ACCi treatment and distinct bioenergetics profiles, we investigated their cellular bioenergetics adaptation in response to ACCi treatment over time. OCR and ECAR were measured at 72 and 144 hours in the presence and absence of ACCi. These time points were chosen to compliment the time points used in the cellular proliferation assay. When U87 and U87 EGFRvIII cells were proliferating (in the presence of vehicle from 0 to 144 hours); both cell lines increased their intrinsic cellular respiration by at least 2 fold (S3B Fig). Interestingly, U87 EGFRvIII cellular ECAR increased by 5.4 fold between 0 and 144 hours (11468 ± 402.8 mpH/min/mg protein versus 2139 ± 96.8 mpH/min/mg protein) while U87 cellular ECAR only increased by 2.7 fold ((5161 ± 443.5 mpH/min/mg protein versus 1930 ± 85.4 mpH/min/mg protein, S3B Fig) revealing a greater proliferation capacity for U87 EGFRvIII cells compared to U87 control cells. After 144 hours of treatment with ACCi, the bioenergetics of U87 cells in the presence of glucose was only slightly affected with no change in OCR and only a modest decrease in ECAR (Fig 3D). Strikingly, U87 EGFRvIII OCR and ECAR were significantly increased by chronic ACCi treatment, despite a reduction in their cellular proliferation capacity. Thus the bioenergetic profile of U87 cells is relatively insensitive to ACCi-mediated metabolic changes. On the contrary, the bioenergetic profile of U87 EGFRvIII cells demonstrated a response to chronic ACCi treatment through an increase in both the OCR and ECAR. Chronic ACCi treatment altered mitochondrial health by decreasing both the maximal mitochondrial respiration (Fig 3F, upper panel) and mitochondrial respiratory control ratio (RCR) of U87 and U87 EGFRvIII cells (Fig 3F, middle panel). However, the maximal stimulated FCCP OCR (fold over baseline) and RCR in U87 EGFRvIII cells were more extensively reduced by chronic ACCi treatment (Fig 3F, upper and middle panels and S5 Table). The precise bioenergetics values can be found in S5 Table. Functional mitochondrial studies were complemented with the assessment of mitochondrial markers expression such as citrate synthase (CS), mitochondrial encoded NADH-ubiquinone oxidoreductase chain 1 (MT-ND1) and ATP synthase 6 (MT-ATP6). Indeed, CS and MT-ND1 transcript levels were decreased in U87 EGFRvIII cells by approximately 40% (0.61 ± 0.17 fold) and 45% (0.55 ± 0.14 fold) respectively indicating that chronic ACCi treatment impacted the mitochondrial transcriptional profile in these cells (Fig 3F, lower panel).

**Fig 4:**
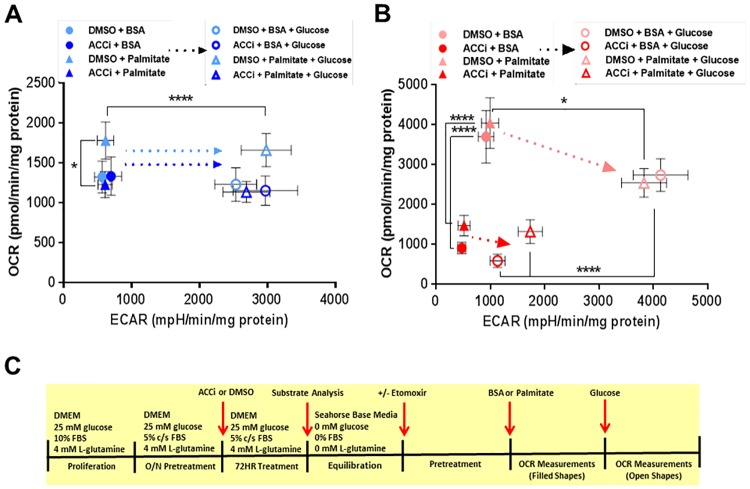
Inhibition of ACC alters the metabolic response of U87 and U87 EGFRvIII cells to fatty acids and glucose. (A) Oxygen Consumption Rate (OCR) and Extracellular Acidification Rate (ECAR) values for U87 cells after treatment with 30 μM ACCi or DMSO control for 72 hours followed by acute treatment with BSA or 100 μM palmitate and 10 mM glucose as assessed by Seahorse XF96e system. Data are expressed as pmol/min/mg protein (OCR) and mpH/min/mg protein (ECAR). Each data point represents mean +/- sem, n = 3. * p<0.05, ****p<0.0001. (B) Oxygen Consumption Rate (OCR) and Extracellular Acidification Rate (ECAR) values for U87 EGFRvIII cells after treatment with 30 μM ACCi or DMSO control for 72 hours followed by acute treatment with BSA or 100 μM palmitate and 10 mM glucose as assessed by Seahorse XF96e system. Data are expressed as pmol/min/mg protein (OCR) and mpH/min/mg protein (ECAR). Each data point represents mean +/- sem, n = 3. * p<0.05, ****p<0.0001. (C) Experimental timeline and media compositions for Figs 4A and 4B.

Finally, we addressed the bioenergetic capacity of U87 and U87 EGFRvIII cells to respond to metabolic challenges such as glucose and palmitate. Seventy-two hours of treatment with ACCi did not alter the basal OCR and ECAR of U87 cells. Treatment with palmitate significantly increased the OCR of U87 cells, however this effect was not observed upon chronic ACCi treatment (Fig 4A). Subsequent addition of excess glucose led to a significant increase in ECAR while OCR was maintained, irrespective of chronic ACCi treatment. Thus, chronic ACCi treatment blunted the capacity of U87 cells to respond to fatty acids (palmitate-mediated OCR) but did not impair the ability of U87 cells to respond to glucose stimulation (glucose-mediated ECAR) in any of the conditions tested (Fig 4A). In contrast to the U87 control cell line, U87 EGFRvIII cells OCR was not significantly upregulated by palmitate treatment and chronic ACCi treatment drastically downregulated respiration in both the presence and absence of palmitate (Fig 4B). After addition of excess glucose, U87 EGFRvIII cellular OCR decreased while the ECAR significantly increased. The shift to a higher ECAR was significantly blunted upon chronic treatment with ACCi (Fig 4B). Thus, U87 and U87 EGFRvIII cell lines differ by their metabolic response to fatty acids and by their capacity to respond to glucose challenge upon chronic ACCi treatment.

### Chronic inhibition of ACC induces apoptosis in U87 and U87 EGFRvIII cells

**Fig 5:**
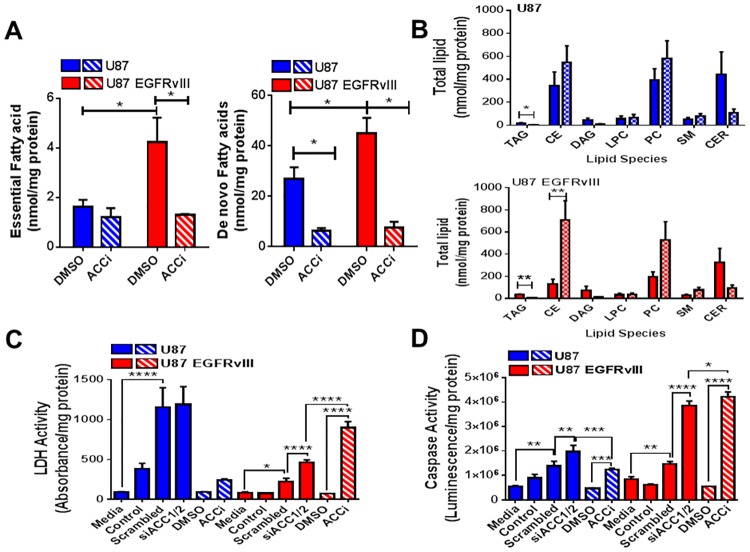
Inhibition of ACC affects lipids content, cell viability and triggers U87 and U87 EGFRvIII cell death. (A) Essential fatty acid (C18:2), left panel, and de novo fatty acids (C16:0, C16:1 and C18:0), right panel, in U87 and U87 EGFRvIII cells treated with 30 μM ACCi or DMSO control for 144 hours as assessed by UPLC MS/MS. Data are represented as nmol/mg protein. Each data point represents mean +/- sem, n = 3. *p<0.05. (B) Effect of 144 hours treatment with 30 μM ACCi (patterned bars) or DMSO control (solid bars) on total cellular lipid species in U87 (top panel) and U87 EGFRvIII (lower panel) cells as assessed by UPLC-MS/MS. Data are expressed as nmol/mg protein. Each data point represents mean +/- sem, n = 3. *p<0.05, **p<0.01. (C) Cellular cytotoxicity after 144 hours treatment with either 30 μM ACCi, DMSO control, a combination of siRNAs targeted to ACC1 and ACC2 (siACC1/2) or scrambled siRNA control on U87 and U87 EGFRvIII as assessed by measurement of lactate dehydrogenase release into the assay media. Control samples were treated with transfection reagent only. Media samples were not treated with transfection reagent or DMSO. Data are represented as absorbance units per milligram of total protein per well. Each data point represents mean +/- sem, n = 3. *p<0.05, ****p<0.0001. (D) Cellular caspase activity after 144 hours treatment with either 30 μM ACCi, DMSO control, a combination of siRNAs targeted to ACC1 and ACC2 (siACC1/2) or scrambled siRNA control on U87 and U87 EGFRvIII cells as assessed by measurement of caspase 3/7 activity. Control samples were treated with transfection reagent only. Media samples were not treated with transfection reagent or DMSO. Data are represented as luminescence per milligram of total protein per well. Each data point represents mean +/- sem, n = 3. *p<0.05, **p<0.01, ****p<0.0001.

To further dissect chronic ACCi treatment on cellular lipids content, total triglycerides were measured by UPLC-MS/MS in the presence of vehicle (DMSO) or ACCi for both U87 and U87 EGFRvIII cells. The total triglyceride content of U87 cells remained quite stable during the treatment time course in the presence of DMSO but was significantly decreased after 72 hours of treatment with ACCi (31 ± 6.6 nmol/mg protein versus 7.3 ± 2.0 nmol/mg protein, S4A Fig). Surprisingly, triglyceride levels in U87 EGFRvIII cells were only slightly altered during the first 120 hours of ACCi treatment and were significantly decreased by approximately 87% after 144 hours of treatment with ACCi (36 ± 5nmol/mg protein DMSO versus 4.7 ± 1nmol/mg protein), (S4B Fig). Given the changes that we observed in total triglycerides between the U87 and U87 EGFRvIII cells, we next compared the levels of dietary-derived essential fatty acid C18:2 to de novo synthesized fatty acids C16:0, C16:1 and C18:0. U87 EGFRvIII cells displayed a 2.6 fold higher basal level of C18:2 compared to U87 cells (4.2 ± 1 nmol/mg protein versus 1.6 ± 0.3 nmol/mg protein, Fig 5A, left panel). After 144 hours of treatment with ACCi, the level of C18:2 fatty acids was similar between U87 EGFRvIII and U87 cells (1.3 ± 0.03 nmol/mg protein versus 1.2 ± 0.4 nmol/mg protein). U87 EGFRvIII cells had approximately 1.7 fold higher concentration of de novo synthesized fatty acids compared to U87 cells under basal DMSO treatment conditions (45.1 ± 6.1 nmol/mg protein versus 26.8 ± 4.7 nmol/mg protein, Fig 5A, right panel). Treatment with ACCi for 144 hours resulted in a dramatic decrease of de novo synthesized fatty acids levels in both U87 and U87 EGFRvIII cells (6.3 ± 1.1 nmol/mg protein versus 7.7 ± 2.3 nmol/mg protein).

Given our observations of the differential effects of dual ACC1/2 inhibition on DNL, proliferation and total triglyceride content after 72 hours in U87 and U87 EGFRvIII cells, we investigated changes to the transcriptional profiles of key lipogenic and stress-related genes (sterol regulatory element-binding protein 1c (SREBP-1c), ATP citrate lyase (ACLY), fatty acid synthase (FASN), stearoyl-CoA desaturase-1 (SCD1) and prostaglandin-endoperoxide synthase 1 (PTGS1)). After 72 hours of chronic ACC inhibition, U87 cells displayed significantly increased expression of FASN and SCD-1, giving 2.3 ± 0.7 and 4 ± 0.6 fold increases respectively (S4C Fig). U87 EGFRvIII cells had modest changes in SREBP-1c and SCD1 but displayed a significant increase in the expression of PTGS1 after 72 hours of ACC inhibition (S4D Fig).

Moreover, chronic ACC inhibition significantly decreased the relative contribution of triacylglycerides (TAG) to the lipids pools and modestly decreased the relative contribution to diacylglycerides (DAG) and ceramides (CER) in both U87 and U87 EGFRvIII cells, Fig 5B, upper and lower panels. Similarly to the modest changes in DAG and CER, chronic ACC inhibition trended to increase phosphatidylcholine (PC) and cholesterol esters (CE) in U87 EGFRvIII cells but not in U87 cells, Fig 5B, upper and lower panels.

Finally, since ACC inhibition affected DNL, proliferation and mitochondrial health, we next evaluated its impact on the expression of select oncogenes. Chronic treatment with ACCi for 144 hours significantly induced MYC, JUN and REL while ABL2, RAF1 and USF2 expression remained similar to DMSO control treated U87 cells (S4E Fig). In contrast ABL2, SRC, RAF1 and E2F1 transcripts were significantly upregulated in U87 EGFRvIII cells in response to ACCi treatment (S4F Fig). To investigate the mechanism by which ACCi treatment reduced U87 EGFRvIII cell number, cytotoxicity (lactate dehydrogenase release) and apoptosis (caspase 3/7 activity) assessments were performed after 144 hours of either ACCi treatment or dual ACC1/2 siRNA knockdown. ACC inhibition increased both LDH release and caspase 3/7 activity in U87 EGFRvIII cells by either siRNA technology or our chemical tool, ACCi (Fig 5C and 5D). LDH release from U87 cells was only slightly increased by ACCi treatment (Fig 5C). More importantly, chronic dual ACC1/2 inhibition elevated the release of LDH (Fig 5C) and caspase activity (Fig 5D) in U87 EGFRvIII cells. Thus, chronic ACC inhibition reduced U87 EGFRvIII cellular number by notably triggering cell death.

## Discussion

Glioblastoma (GBM) is among the most resistant cancers to radiation and chemotherapy, which also makes it one of the most lethal and largely untreatable with a median survival after diagnosis of approximately 15 months [28]. Epithelial growth factor receptor (EGFR) mutations are the most commonly found mutations in GBM tumors with approximately 50% of EGFR-mutated GBM tumors harboring the EGFRvIII mutation [29]. The EGFRvIII mutation has been shown *in vitro* to increase U87 GBM cells dependence on de novo fatty acid synthesis [26]. Current treatment options consist of surgical removal and a combination of chemo- and radiation therapy [28] which highlights the need to explore other therapeutic options.

Importance of DNL for cellular proliferation and survival have been reported in other forms of cancer such as lung [9], colon [30], prostate [10], and breast [11], enforcing the important role of lipids synthesis for the proliferation of certain cancers. Recently, Svensson *et al*. also provided genetic and pharmacological evidence for the role of ACC in lung tumor growth [31]. Using both *in vitro* and preclinical models, they reported that ACC inhibition (pharmacologically and genetically) reduced *de novo* lipids synthesis and decreased the growth and viability of non-small-cell lung cancer cells. We demonstrated that inhibition of ACC in two human glioblastoma cell lines, U87 and U87 EGFRvIII, resulted in a similar impairment of ^14^C-acetate incorporation into neutral lipids, a marker of de novo lipogenesis (DNL), while U87 EGFRvIII cellular proliferation was more sensitive to ACC inhibition than U87 cellular proliferation. Thus the capacity of ACCi to inhibit ^14^C-acetate uptake in any cancer cells is not predictive of its capacity to inhibit cellular proliferation. As opposed to the drastic decrease in the total triacylglycerides (TAG) content in U87 cells (S4A Fig), chronic ACCi treatment interestingly shifted the total relative contribution of various lipid pools in U87 EGFRvIII cells. Indeed, the relative contribution of triacylglycerides (TAG), diacylglycerides (DAG) and ceramides (CER) tended to decrease while cholesterol esters (CE) contribution to the total lipids pool was increased upon chronic ACCi treatment (Fig 5B). Consequences of this shift on cellular bioenergetics, mitochondrial health and cell proliferation remains to be elucidated.

Inhibition of ACC with a dual small molecule inhibitor as well as with dual siRNA ACC1/2 knockdown not only blunted de novo lipogenesis but also dramatically impaired U87 EGFRvIII cellular proliferation and viability. We investigated the mechanism of cell death and demonstrated that U87 EGFRvIII cells underwent apoptosis. It has been previously shown that overexpression of E2F1 in glioma cell lines induced apoptosis through the activation of caspases in these cell lines [32]. Moreover, chronic inhibition of ACC in the U87 EGFRvIII cells for 144 hours resulted in the upregulation of E2F1 gene expression, while this did not occur in the U87 control cells (S4E and S4F Fig). These data correlate nicely with the increase in caspase signal after ACCi treatment in U87 EGFRvIII cells (Fig 5D). After 144 hours of ACC inhibition, U87 cells exhibited increased levels of MYC gene expression, whereas U87 EGFRvIII cells did not (S4E and S4F Fig). It is known that MYC controls many glycolytic genes and has been shown to enhance aerobic glycolysis, cell proliferation rates and anabolic processes [33,34]. These anabolic processes also require mitochondrial produced substrates [33] and the induction of MYC in cells has been shown to increase mitochondrial oxygen consumption and mitochondrial mass [34]. Interestingly, the oxygen consumption rate of U87 cells did not significantly change after 144 hours of ACCi treatment (Fig 3D) and U87 cells had higher expression of mitochondrial genes compared to U87 EGFRvIII cells both at basal (S5A Fig) and after 144 hours of ACCi treatment (Fig 3F, lower panel).

Under basal conditions, SRC and RAF1 mRNA expression was increased in U87 cells while MYC and JUN gene expression was elevated in U87 EGFRvIII cells (S5B Fig). Notably, after 72 hours of ACC inhibition, we observed an overall downregulation or maintenance of selected oncogene expression in U87 cells (S6A Fig). However, most of the selected oncogenes expression in U87 EGFRvIII cells were significantly increased, notably MYC, after 72 hours of ACCi treatment (S6B Fig). PTGS1 was virtually undetected in U87 and U87 EGFRvIII cells under basal conditions (S5C Fig) but was significantly upregulated in U87 EGFRvIII cells after 72 hours of ACCi treatment (S4D Fig). PTGS1, also known as COX-1, is known to play a role in prostaglandin synthesis and has been shown to be linked to TNF-related apoptosis-inducing ligand (TRAIL)-induced apoptosis in a breast carcinoma cell line, MDA-MB-453 [35]. This result, paired with increased U87 EGFRvIII cellular caspase activity demonstrated that ACCi treatment triggered transcriptional remodeling and significant cellular metabolic stress.

The bioenergetics profiles of U87 and U87 EGFRvIII cell lines revealed cell-specific metabolic rates. For instance, under basal conditions and after 72 hours, U87 EGFRvIII cells displayed higher rates of respiration (OCR) and extracellular acidification (ECAR) than U87 cells, most likely to match their higher energy demands for rapid proliferation. The increased OCR in U87 EGFRvIII cells appears to be dependent on exogenous lipids as it was not maintained upon chronic exposure to delipidated serum. However the difference in ECAR between U87 and U87 EGFRvIII cells, which reflects the combination of extracellular milieu acidification by CO_2_ released through increased mitochondrial activity as well as by lactate production and subsequent media acidification mediated by glycolysis, became greater over time. Surprisingly, U87 EGFRvIII cells displayed a greater maximal mitochondrial oxidation capacity under basal conditions (high glucose and amino acids) compared to U87 cells. This is not likely due to increased mitochondria mass or number as shown by similar expression of citrate synthase (S5A Fig) or by increased CPT1A activity (Fig 3A and 3B).

When chronically incubated in the presence of ACCi, we observed that the cellular metabolic response of U87 EGFRvIII cells was time dependent. After 72 hours of ACCi treatment, the metabolic rate was unchanged in U87 cells but was decreased in U87 EGFRvIII cells in the presence of glucose, as demonstrated by OCR and ECAR (Fig 4A and 4B, open circles). However, after 144 hours of ACCi treatment, U87 EGFRvIII cells increased their metabolic rate in response to ACCi in presence of glucose (Fig 3D). Furthermore, chronic 144 hour ACCi treatment significantly decreased both U87 and U87 EGFRvIII cellular RCR (Fig 3F, middle panel), indicating a loss of ATP synthase controlled respiration. Indeed, a lower RCR could be the force driving the increased metabolic rate of the U87 EGFRvIII cells but it wouldn’t be possible without the increased endogenous/exogenous lipid-mediated oxidation rate (Fig 3B). As our ACCi molecule does not display intrinsic acute uncoupler activity (data not shown), we speculate that lower RCR may have resulted from either lipotoxicity or reactive oxygen species production due to chronic ACCi treatment while we can’t exclude at this time point a potential uncoupling effect from an ACCi intermediate metabolite. While the mechanism by which chronic ACCi treatment led to leakage of mitochondrial energy remains to be elucidated, inefficiency to produce ATP may have participated in the reduction of U87 EGFRvIII cellular proliferation as well as in the triggering of apoptosis. When chronically treated with ACCi for 144 hours, U87 cells remained able to respond to a bolus of glucose in the presence or absence of palmitate (Fig 4A). On the other hand, addition of either palmitate or excess of glucose did not strikingly improve U87 EGFRvIII OCR and ECAR after chronic treatment with ACCi, indicating a loss of metabolic flexibility and most likely contributing to the loss of cellular viability. Thus, a mechanism suppressing de novo lipogenesis and increasing the rate of mitochondrial substrate oxidation such as ACC inhibition could serve as a potential anti-cancer therapy.

## Conclusion

Altogether, we have demonstrated that ACC inhibition, by reducing DNL and increasing mitochondrial oxidation rates, may have therapeutic utility for the suppression of aggressive GBM tumor growth, and that this mechanism warrants further investigation as a potential cancer therapy.

## Supporting Information

S1 Fig Comparison of cellular proliferation of U87 and U87 EGFRvIII cells. Cellular proliferation of U87 and U87 EGFRvIII cells was assessed after 48 hours by measurement of luciferase signal, reflecting ATP content. This time point corresponds to the time at which the inhibition of DNL was measured. Each data point represents mean +/- sem, n = 3–4. *p<0.05, ****p<0.0001.
(TIF)

S2 Fig Assessment of siRNA knockdown of ACC1 and ACC2 (siACC1/2) after 72 hours in U87 and U87 EGFRvIII cells. (A) Knockdown of mRNA expression for ACC1 (left panel) and ACC2 (right panel) after 72 hours treatment with a combination of siRNAs targeted to ACC1 and ACC2 (siACC1/2) or scrambled control siRNA as assessed by qPCR. Data are normalized to housekeeping genes *hPPIA* and *hTBP*. * p<0.05, *** p<0.001. Each data point represents mean +/- sem, n = 3. (B) Measurement of ACC1 and ACC2 protein expression after 72 hours treatment with a combination of siRNAs targeted to ACC1 and ACC2 (siACC1/2) or scrambled control siRNA as assessed by western blot. Alpha tubulin was used as a loading control. Representative blots shown from n = 3 experiments. (C) Quantification of western blot protein expression of ACC1 (left panel) and ACC2 (right panel) after 72 hours treatment with a combination of siRNAs targeted to ACC1 and ACC2 (siACC1/2) or scrambled control siRNA. Calculations were based on volume intensity of the bands. Data are normalized to α-tubulin loading control. **** p<0.0001. Each data point represents mean +/- sem, n = 3. (D) Expression of ACC1 mRNA at basal and over time with treatment with 30 μM ACCi or DMSO control in U87 and U87 EGFRvIII cell lines as assessed by qPCR. Data are represented as fold change from DMSO control. Each data point represents mean +/- sem, n = 3. (E) Expression of ACC2 mRNA at basal and over time with treatment with 30 μM ACCi or DMSO control in U87 and U87 EGFRvIII cell lines as assessed by qPCR. Data are represented as fold change from DMSO control. Each data point represents mean +/- sem, n = 3.
(TIF)

S3 Fig CPT1A expression and metabolic activity in U87 and U87 EGFRvIII cell lines. (A) Expression of CPT1A mRNA over time in the presence of 30 μM ACCi or DMSO control in both U87 and U87 EGFRvIII cell lines as assessed by qPCR. Data are normalized to the expression of housekeeping genes. Each data point represents mean +/- sem, n = 3. * p<0.05. (B) Oxygen Consumption Rate (OCR) and Extracellular Acidification Rate (ECAR) values for U87 and U87 EGFRvIII cells over time as assessed by Seahorse XF96e system. Data are expressed as pmol/min/mg protein (OCR) and mpH/min/mg protein (ECAR). Each data point represents mean +/- sem, n = 3. *** p<0.001, ****p<0.0001.
(TIF)

S4 Fig Changes in U87 and U87 EGFRvIII cellular gene expression and lipids profile upon ACCi treatment. (A) Total triglyceride levels in U87 cells treated with 30 μM ACCi or DMSO control over time as assessed by UPLC-MS/MS. Data are represented as nmol/mg protein. Each data point represents mean +/- sem, n = 3. *p<0.05. (B) Total triglyceride levels in U87 EGFRvIII cells treated with 30 μM ACCi or DMSO control over time as assessed by UPLC-MS/MS. Data are represented as nmol/mg protein. Each data point represents mean +/- sem, n = 3. **p<0.01. (C) Expression of lipogenic genes after treatment with 30 μM ACCi or DMSO control for 72 hours in U87 cells as assessed by qPCR. Data are expressed as fold change from DMSO control. Each data point represents mean +/- sem, n = 3–4 experiments. ***p<0.001, **** p<0.0001. (D) Expression of lipogenic genes after treatment with 30 μM ACCi or DMSO control for 72 hours in U87 EGFRvIII cells as assessed by qPCR. Data are expressed as fold change from DMSO control. Each data point represents mean +/- sem, n = 3–4 experiments. **** p<0.0001. (E) Changes in oncogene mRNA expression after 144 hours of treatment with 30 μM ACCi or DMSO control in U87 cells as assessed by qPCR. Data are expressed as fold change from DMSO control. Each data point represents mean +/- sem, n = 3. *p<0.05, **p<0.01. (F) Changes in oncogene mRNA expression after 144 hours of treatment with 30 μM ACCi or DMSO control in U87 EGFRvIII cells as assessed by qPCR. Data are expressed as fold change from DMSO control. Each data point represents mean +/- sem, n = 3. *p<0.05, ****p<0.0001.
(TIF)

S5 Fig U87 and U87 EGFRvIII cells basal mitochondrial, oncogene and lipogenic gene expression assessment. (A) Basal mitochondrial gene expression as assessed by qPCR. Data are normalized to the expression of housekeeping genes expression. Each data point represents mean +/- sem, n = 3–7. **p<0.01, ***p<0.001. (B) Basal oncogene gene expression as assessed by qPCR. Data are normalized to the expression of housekeeping genes expression. Each data point represents mean +/- sem, n = 4–7. *p<0.05, **p<0.01, ****p<0.0001. (C) Basal lipogenic gene expression as assessed by qPCR. Data are normalized to the expression of housekeeping genes expression. Each data point represents mean +/- sem, n = 3–7. **p<0.01.
(TIF)

S6 Fig Change in U87 and U87 EGFRvIII cells oncogene expression upon ACCi treatment. (A) mRNA expression of oncogenes after 72 hours of treatment with 30 μM ACCi or DMSO control in U87 cells as assessed by qPCR. Data are expressed as fold change from DMSO control. Each data point represents mean +/- sem, n = 3–4.*p<0.05. (B) mRNA expression of oncogenes after 72 hours of treatment with 30 μM ACCi or DMSO control in U87 EGFRvIII cells as assessed by qPCR. Data are expressed as fold change from DMSO control. Each data point represents mean +/- sem, n = 3–4. ***p<0.001, ****p<0.0001.
(TIF)

S1 Table Media composition for individual Seahorse experiments. (TIF)

S2 Table Comparison of amino acids composition in DMEM and Seahorse media. (TIF)

S3 Table Taqman probes used for qPCR analyses. (TIF)

S4 Table A: **Antibodies used for western blot analyses**. B: **siRNAs used for knockdown experiments**.
(TIF)

S5 Table Summary of OCR values from bioenergetics experiment after 144 hours treatment with 30 μM ACCi or DMSO control. (TIF)
