# Supplementary figures and images for: Inhibition of Acetyl-CoA Carboxylase 1 (ACC1) and 2 (ACC2) Reduces Proliferation and De Novo Lipogenesis of EGFRvIII Human Glioblastoma Cells
Source: PLoS One. 2017 Jan 12;12(1):e0169566. doi: 10.1371/journal.pone.0169566 (PMC5231342; doi:10.1371/journal.pone.0169566)

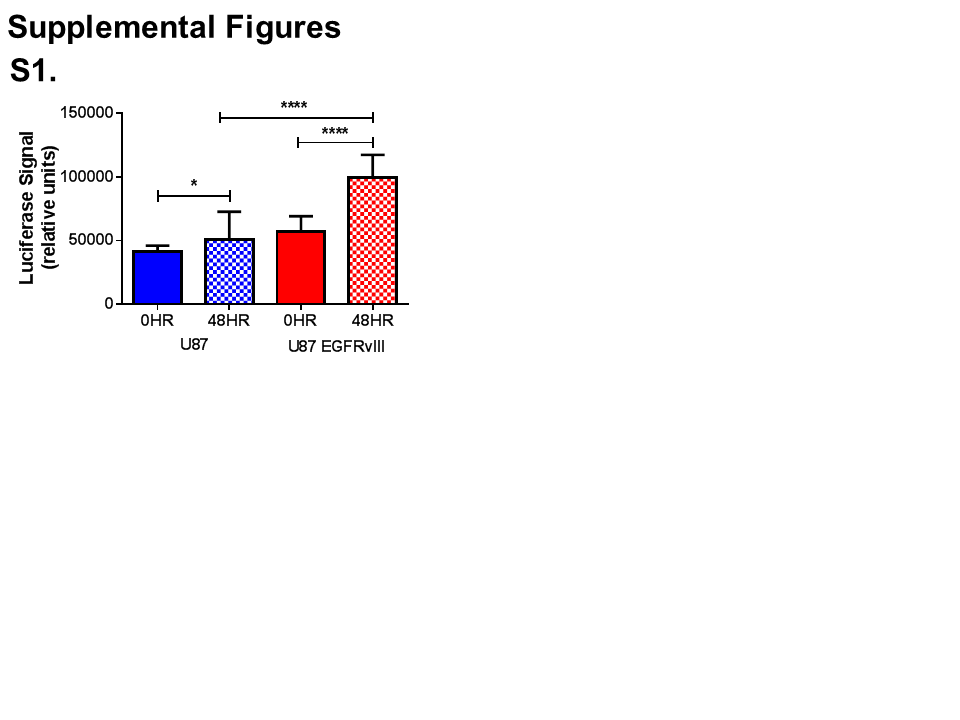

Supplement: S1 Fig — Cellular proliferation of U87 and U87 EGFRvIII cells was assessed after 48 hours by measurement of luciferase signal, reflecting ATP content. This time point corresponds to the time at which the inhibition of DNL was measured. Each data point represents mean +/- sem, n = 3–4. *p<0.05, ****p<0.0001. (TIF) [file pone.0169566.s001.tif]

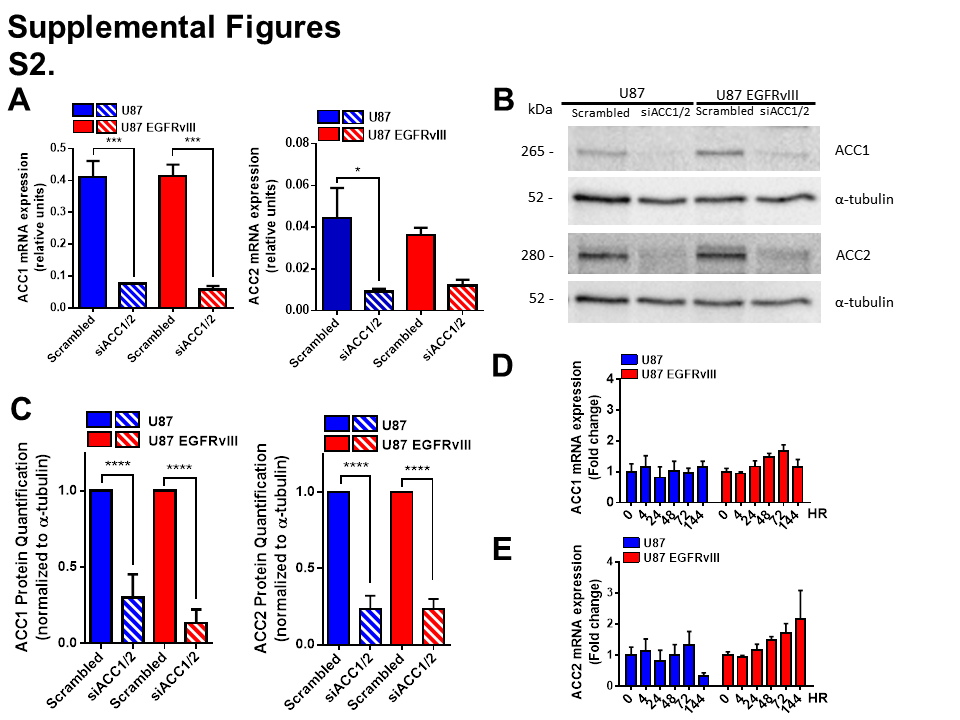

Supplement: S2 Fig — (A) Knockdown of mRNA expression for ACC1 (left panel) and ACC2 (right panel) after 72 hours treatment with a combination of siRNAs targeted to ACC1 and ACC2 (siACC1/2) or scrambled control siRNA as assessed by qPCR. Data are normalized to housekeeping genes hPPIA and hTBP. * p<0.05, *** p<0.001. Each data point represents mean +/- sem, n = 3. (B) Measurement of ACC1 and ACC2 protein expression after 72 hours treatment with a combination of siRNAs targeted to ACC1 and ACC2 (siACC1/2) or scrambled control siRNA as assessed by western blot. Alpha tubulin was used as a loading control. Representative blots shown from n = 3 experiments. (C) Quantification of western blot protein expression of ACC1 (left panel) and ACC2 (right panel) after 72 hours treatment with a combination of siRNAs targeted to ACC1 and ACC2 (siACC1/2) or scrambled control siRNA. Calculations were based on volume intensity of the bands. Data are normalized to α-tubulin loading control. **** p<0.0001. Each data point represents mean +/- sem, n = 3. (D) Expression of ACC1 mRNA at basal and over time with treatment with 30 μM ACCi or DMSO control in U87 and U87 EGFRvIII cell lines as assessed by qPCR. Data are represented as fold change from DMSO control. Each data point represents mean +/- sem, n = 3. (E) Expression of ACC2 mRNA at basal and over time with treatment with 30 μM ACCi or DMSO control in U87 and U87 EGFRvIII cell lines as assessed by qPCR. Data are represented as fold change from DMSO control. Each data point represents mean +/- sem, n = 3. (TIF) [file pone.0169566.s002.tif]

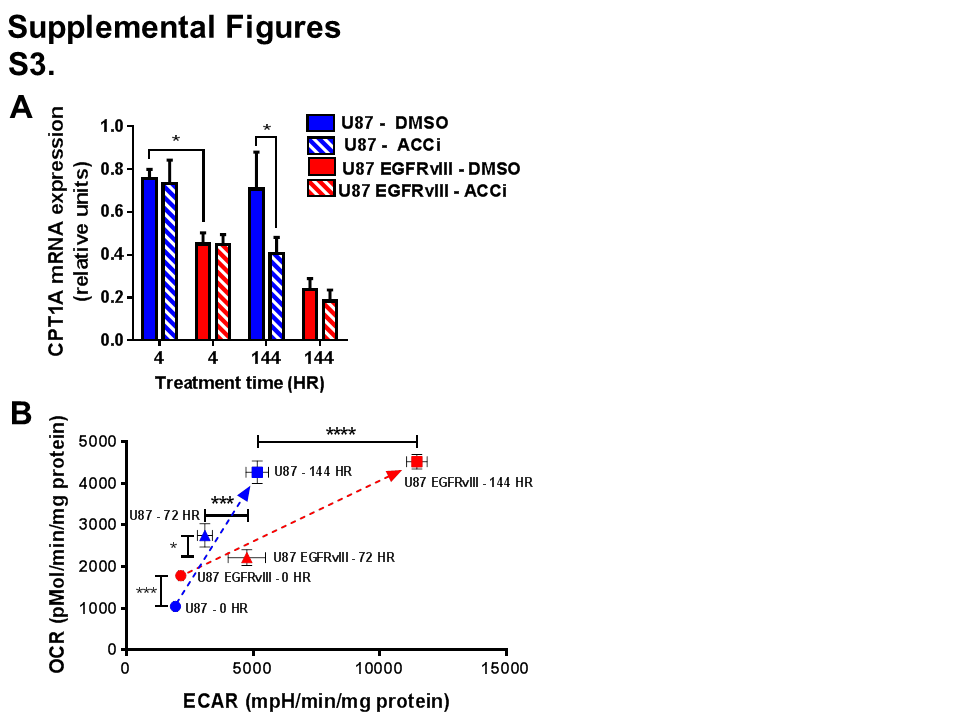

Supplement: S3 Fig — (A) Expression of CPT1A mRNA over time in the presence of 30 μM ACCi or DMSO control in both U87 and U87 EGFRvIII cell lines as assessed by qPCR. Data are normalized to the expression of housekeeping genes. Each data point represents mean +/- sem, n = 3. * p<0.05. (B) Oxygen Consumption Rate (OCR) and Extracellular Acidification Rate (ECAR) values for U87 and U87 EGFRvIII cells over time as assessed by Seahorse XF96e system. Data are expressed as pmol/min/mg protein (OCR) and mpH/min/mg protein (ECAR). Each data point represents mean +/- sem, n = 3. *** p<0.001, ****p<0.0001. (TIF) [file pone.0169566.s003.tif]

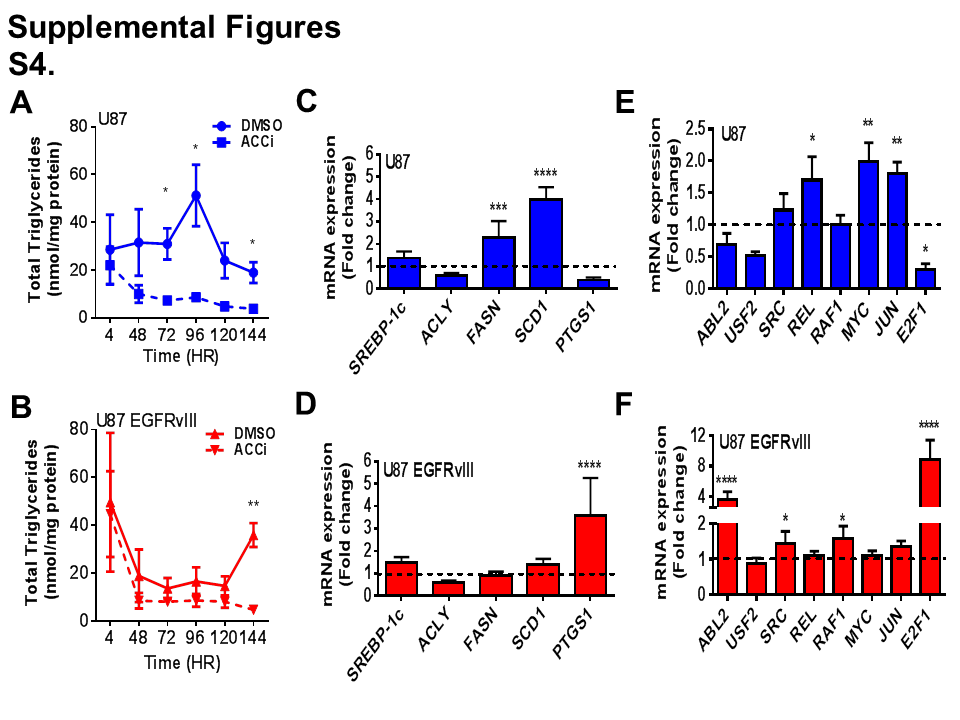

Supplement: S4 Fig — (A) Total triglyceride levels in U87 cells treated with 30 μM ACCi or DMSO control over time as assessed by UPLC-MS/MS. Data are represented as nmol/mg protein. Each data point represents mean +/- sem, n = 3. *p<0.05. (B) Total triglyceride levels in U87 EGFRvIII cells treated with 30 μM ACCi or DMSO control over time as assessed by UPLC-MS/MS. Data are represented as nmol/mg protein. Each data point represents mean +/- sem, n = 3. **p<0.01. (C) Expression of lipogenic genes after treatment with 30 μM ACCi or DMSO control for 72 hours in U87 cells as assessed by qPCR. Data are expressed as fold change from DMSO control. Each data point represents mean +/- sem, n = 3–4 experiments. ***p<0.001, **** p<0.0001. (D) Expression of lipogenic genes after treatment with 30 μM ACCi or DMSO control for 72 hours in U87 EGFRvIII cells as assessed by qPCR. Data are expressed as fold change from DMSO control. Each data point represents mean +/- sem, n = 3–4 experiments. **** p<0.0001. (E) Changes in oncogene mRNA expression after 144 hours of treatment with 30 μM ACCi or DMSO control in U87 cells as assessed by qPCR. Data are expressed as fold change from DMSO control. Each data point represents mean +/- sem, n = 3. *p<0.05, **p<0.01. (F) Changes in oncogene mRNA expression after 144 hours of treatment with 30 μM ACCi or DMSO control in U87 EGFRvIII cells as assessed by qPCR. Data are expressed as fold change from DMSO control. Each data point represents mean +/- sem, n = 3. *p<0.05, ****p<0.0001. (TIF) [file pone.0169566.s004.tif]

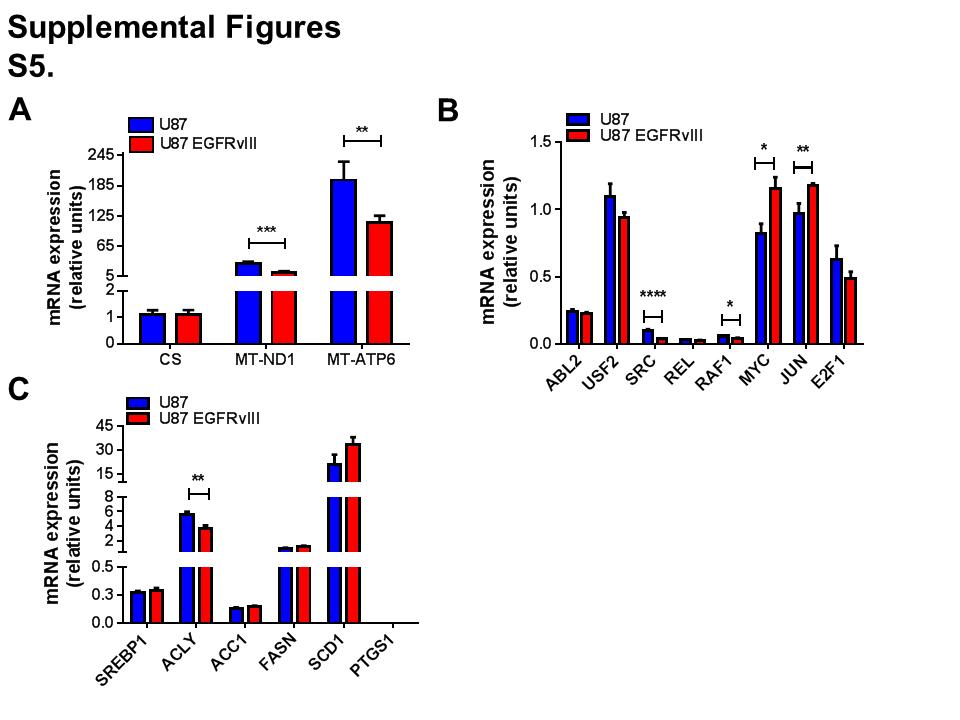

Supplement: S5 Fig — (A) Basal mitochondrial gene expression as assessed by qPCR. Data are normalized to the expression of housekeeping genes expression. Each data point represents mean +/- sem, n = 3–7. **p<0.01, ***p<0.001. (B) Basal oncogene gene expression as assessed by qPCR. Data are normalized to the expression of housekeeping genes expression. Each data point represents mean +/- sem, n = 4–7. *p<0.05, **p<0.01, ****p<0.0001. (C) Basal lipogenic gene expression as assessed by qPCR. Data are normalized to the expression of housekeeping genes expression. Each data point represents mean +/- sem, n = 3–7. **p<0.01. (TIF) [file pone.0169566.s005.tif]

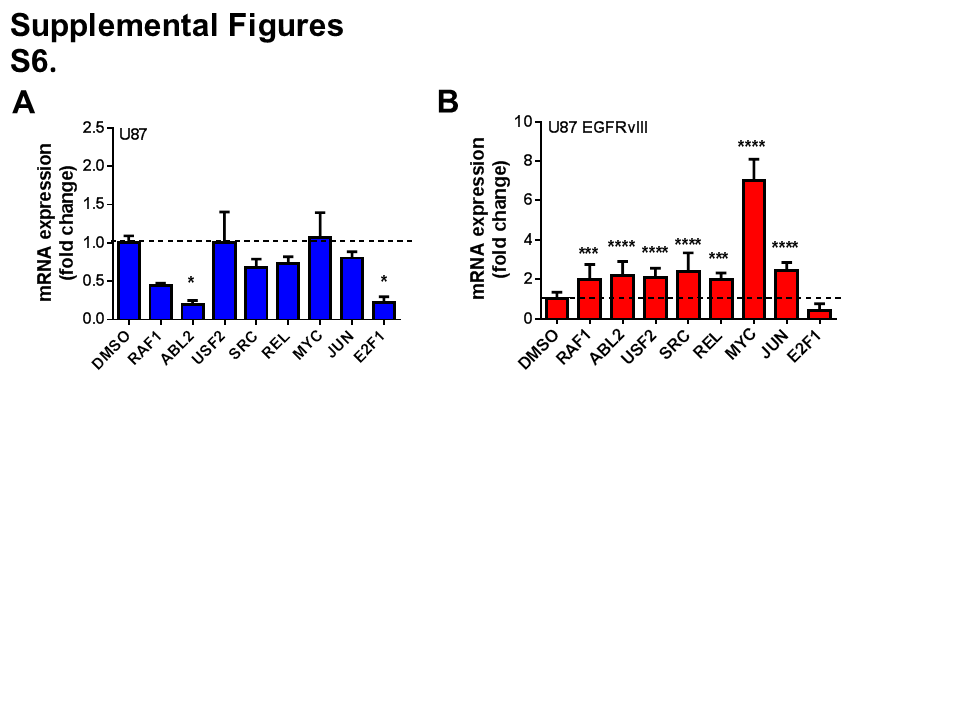

Supplement: S6 Fig — (A) mRNA expression of oncogenes after 72 hours of treatment with 30 μM ACCi or DMSO control in U87 cells as assessed by qPCR. Data are expressed as fold change from DMSO control. Each data point represents mean +/- sem, n = 3–4.*p<0.05. (B) mRNA expression of oncogenes after 72 hours of treatment with 30 μM ACCi or DMSO control in U87 EGFRvIII cells as assessed by qPCR. Data are expressed as fold change from DMSO control. Each data point represents mean +/- sem, n = 3–4. ***p<0.001, ****p<0.0001. (TIF) [file pone.0169566.s006.tif]

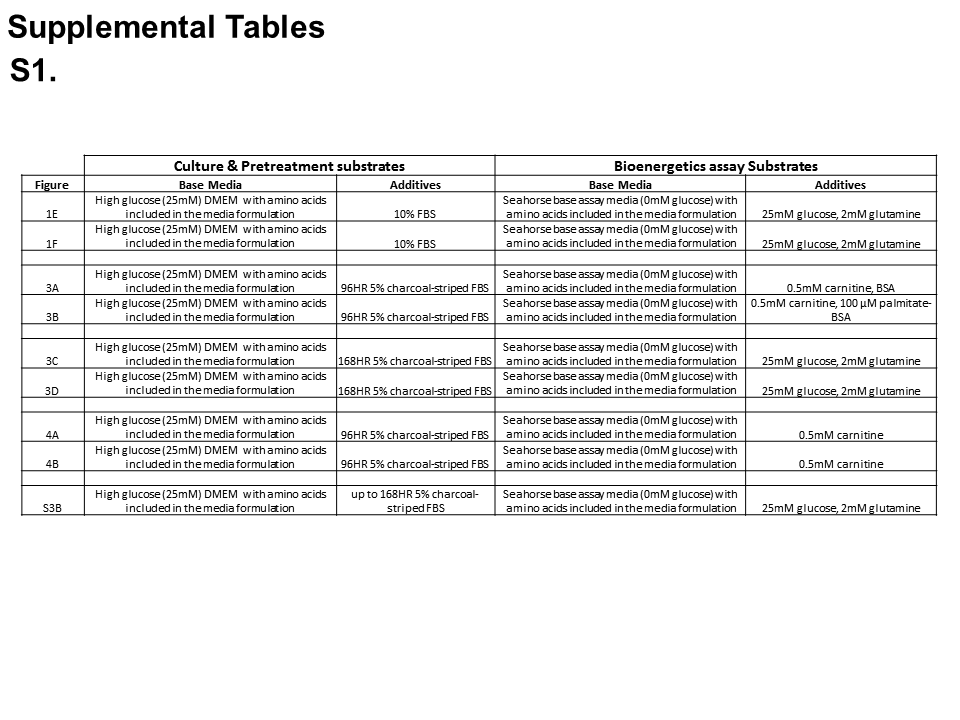

Supplement: S1 Table — (TIF) [file pone.0169566.s007.tif]

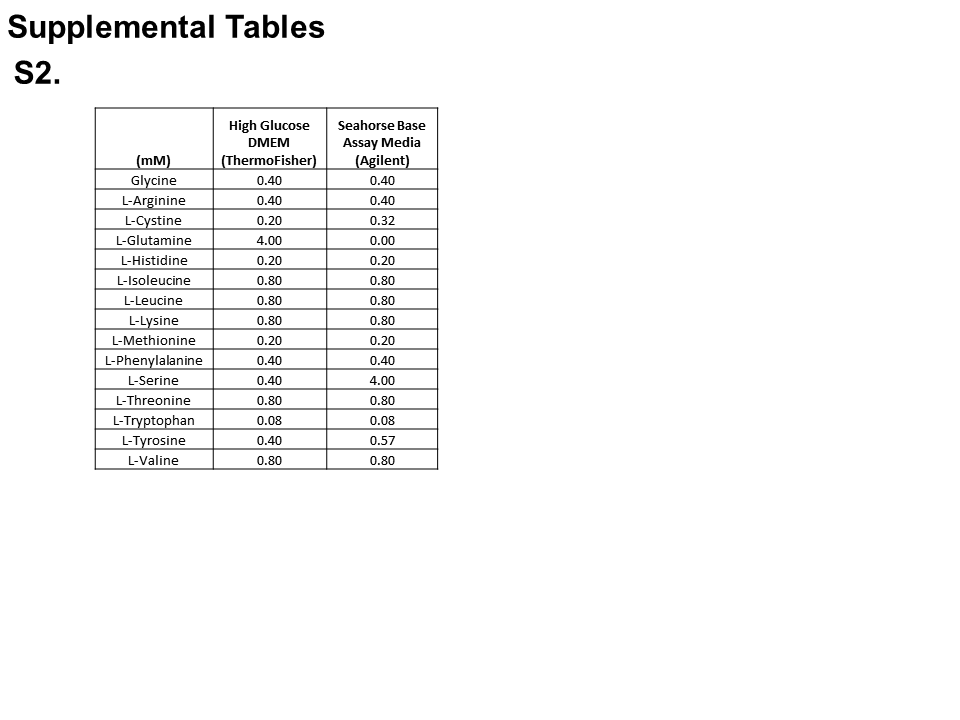

Supplement: S2 Table — (TIF) [file pone.0169566.s008.tif]

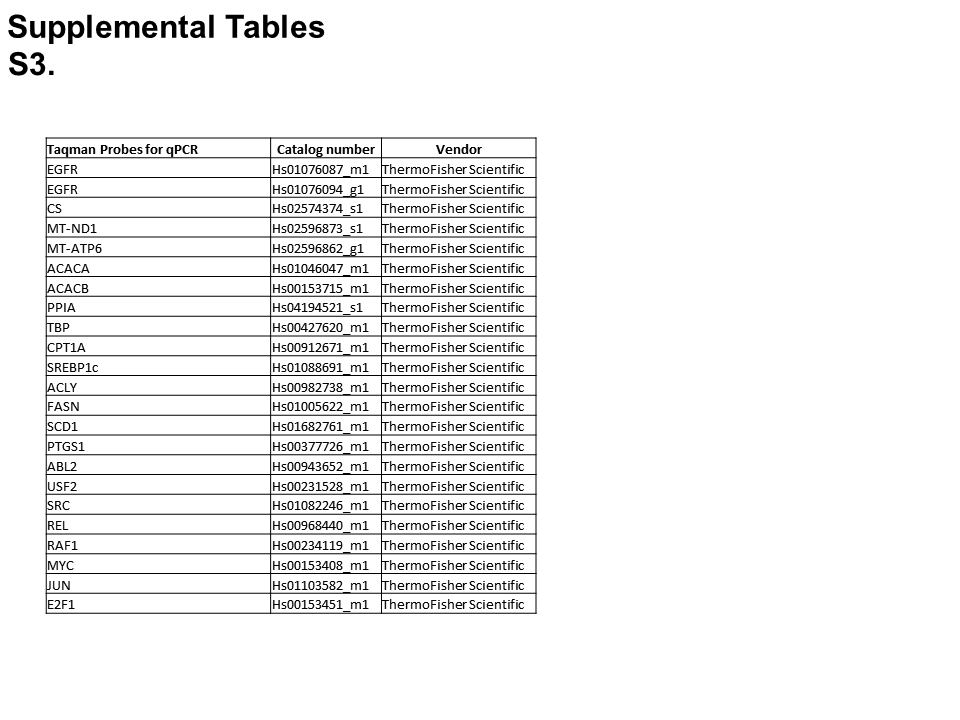

Supplement: S3 Table — (TIF) [file pone.0169566.s009.tif]

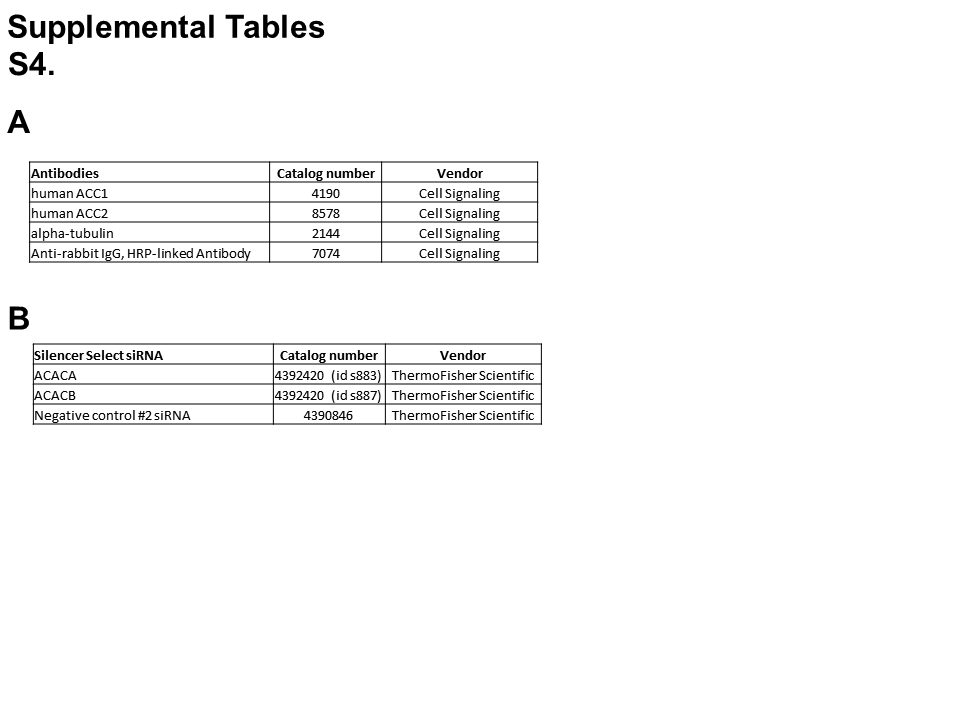

Supplement: S4 Table — A: Antibodies used for western blot analyses. B: siRNAs used for knockdown experiments. (TIF) [file pone.0169566.s010.tif]

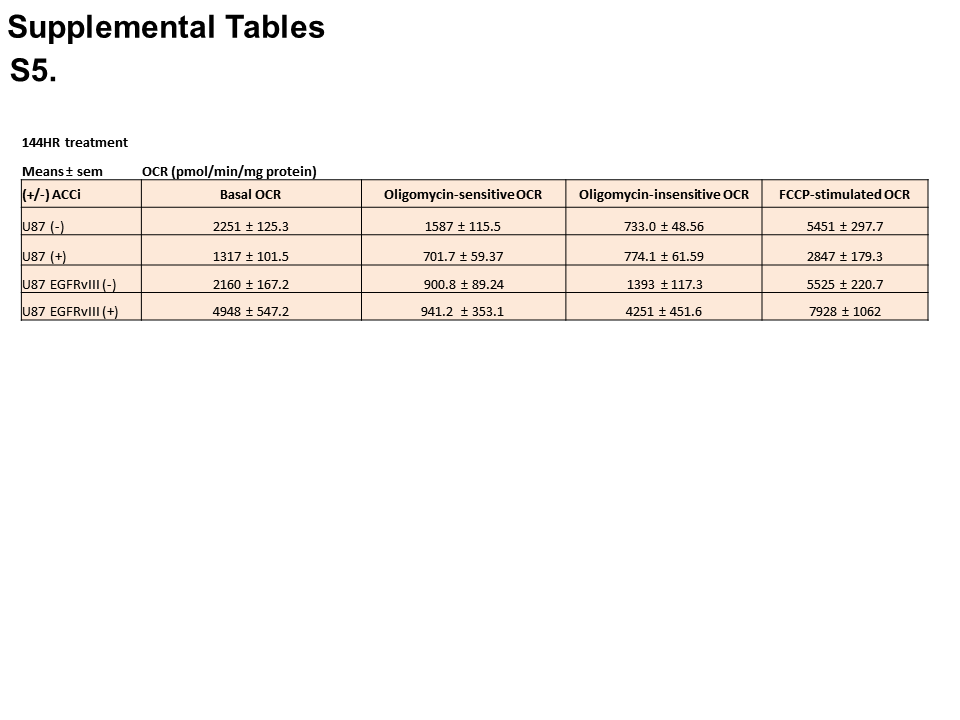

Supplement: S5 Table — (TIF) [file pone.0169566.s011.tif]
